# The Cytoprotective Effects of Baicalein on H_2_O_2_-Induced ROS by Maintaining Mitochondrial Homeostasis and Cellular Tight Junction in HaCaT Keratinocytes

**DOI:** 10.3390/antiox12040902

**Published:** 2023-04-10

**Authors:** Gyeonghyeon Kim, Dong-Wook Han, Jong Hun Lee

**Affiliations:** 1 Department of Food Science and Biotechnology, Gachon University, Seongnam 13120, Republic of Korea; 2 Department of Cogno-Mechatronics Engineering, College of Nanoscience & Nanotechnology, Pusan National University, Busan 46241, Republic of Korea; 3 Bio-IT Fusion Technology Research Institute, Pusan National University, Busan 46241, Republic of Korea

**Keywords:** baicalein, antioxidants, tight junction, mitochondrial homeostasis, Nrf2/NQO-1/HO-1 pathway, HaCaT keratinocytes

## Abstract

Reactive oxygen species (ROS) promote oxidative stress, which directly causes molecular damage and disrupts cellular homeostasis, leading to skin aging. Baicalein, a flavonoid compound isolated from the root of *Scutellaria baicalensis Georgi* has antioxidant, anticancer, anti-inflammatory, and other medicinal properties. We aimed to investigate the protective effect of baicalein on the disruption of tight junctions and mitochondrial dysfunction caused by H_2_O_2_-induced oxidative stress in HaCaT keratinocytes. The cells were pretreated with 20 and 40 µM baicalein followed by treatment with 500 µM H_2_O_2_. The results revealed that baicalein exerted antioxidant effects by reducing intracellular ROS production. Baicalein attenuated the degradation of the ECM (MMP-1 and Col1A1) and the disruption of tight junctions (ZO-1, occludin, and claudin-4). In addition, baicalein prevented mitochondrial dysfunction (PGC-1α, PINK1, and Parkin) and restored mitochondrial respiration. Furthermore, baicalein regulated the expression of antioxidant enzymes, including NQO-1 and HO-1, via the Nrf2 signaling pathway. Our data suggest that the cytoprotective effects of baicalein against H_2_O_2_-induced oxidative stress may be mediated through the Nrf2/NQO-1/HO-1 signaling pathway. In conclusion, baicalein exerts potent antioxidant effects against H_2_O_2_-induced oxidative stress in HaCaT keratinocytes by maintaining mitochondrial homeostasis and cellular tight junctions.

## 1. Introduction

The skin protects the body against external factors by forming a barrier composed of several layers, including the dermis and the epidermis. The skin prevents moisture loss and protects the human body from external stimuli [1]. Based on the determinants of aging, skin aging may be categorized as intrinsic and extrinsic aging. Intrinsic aging occurs as a natural result of physiological changes over time and may occur even in the absence of a specific stimulus [2]. Extrinsic aging, also known as photoaging, is commonly caused by external factors such as solar irradiation and leads to premature aging of the skin. Aging of the skin causes alterations in the skin, such as damage to the skin support system and drying of the skin [3].

Reactive oxygen species (ROS) are generated from diverse sources, including intracellular mitochondria and external stimuli [4,5]. Appropriate levels of ROS transmit intracellular signals and regulate homeostasis. However, excessive ROS induce oxidative stress and damage cells. The accumulation of ROS exacerbates skin aging and inflammation, disrupting cellular tight junctions and mitochondrial dysfunction [6,7,8,9,10].

Cell–cell and cell–matrix adhesions are required to assemble individual epithelial cells into an appropriate form [11]. Cell–matrix adhesion involves the interaction of cells and the extracellular matrix (ECM) and is mediated by multi-protein adhesion structures. The ECM occupies the space between cells and regulates many cellular processes, including tissue growth, differentiation, and homeostasis [12]. ROS cause the degradation of ECM proteins, such as collagen, leading to the loss of skin elasticity [13]. The ECM proteins play an important role in maintaining skin homeostasis. A tight junction is a cell–cell adhesion that maintains cell surface polarity by creating a barrier to regulate the movement of molecules. It also maintains tissue homeostasis by separating regions [14,15]. The tight junction plays an important role in preventing the pathogenesis of several common cutaneous inflammatory and neoplastic conditions [16]. There are several causes that weaken the function of tight junctions. Inflammatory cytokines reduce barrier function by inducing alterations in tight junctions [17,18]. Specifically, ultraviolet radiation (UVR) disrupts the barrier function of the skin by altering the expression and localization of tight junction proteins, leading to the trans-epidermal water loss (TEWL) [19]. Thus, in order to support the function of the skin as a “physical barrier”, it is very important to maintain the function of the tight junction.

Mitochondria play an essential role in aerobic energy production in eukaryotic cells. They produce most of the energy and ATP needed by cells. Mitochondria generate ROS as by-products of aerobic metabolism but are themselves particularly sensitive to ROS-induced oxidative stress [20]. ROS-induced mitochondrial damage leads to the disruption of Ca^2+^ homeostasis, the release of apoptotic proteins, and impaired ATP metabolism [9,21]. Aberrant removal of ROS-induced mitochondrial DNA (mtDNA) mutations may ultimately lead to cell death. Thus, mitochondrial dysfunction may reduce skin function and cause aging [22,23]. A phenomenon used by cells to deal with mitochondrial dysfunction is an intercellular transfer from adjacent cells. Mitochondria or mtDNA are trafficked into the damaged cell to repair the dysfunctional mitochondria [24,25]. Zhang et al. presented a study on the relationship between mitochondrial dynamics and mitophagy in deoxynivalenol-induced porcine intestinal tight junction disorder [26]. Thus, the enhancement of tight junctions may improve mitochondrial function.

Nuclear factor erythroid 2-related factor 2 (Nrf2) is an important regulator of ROS-induced mitochondrial damage [27]. Nrf2 protects cells by maintaining intracellular homeostasis and inducing the expression of oxidative stress-responsive genes [28]. Nrf2 regulates the expression of antioxidant enzymes, such as heme oxygenase-1 (HO-1) and NAD(P)H: quinone oxidoreductase-1 (NQO-1) [29,30]. An effective mechanism by which cells protect themselves is by altering Nrf2 to an equilibrium state, thereby lowering ROS and oxidative stress.

Flavonoids are phenolic compounds that help plants to survive via their antioxidant effects. Flavonoids absorb UVR and reduce damage caused by ROS [31,32]. Baicalein is a flavonoid compound isolated from the root of *Scutellaria baicalensis Georgi.* It has shown an antioxidant effect through the removal of ROS and the enhancement of mitochondrial function [33]. However, its effects on mitochondrial respiration or tight junctions in HaCaT cells have not been reported. In the present study, we hypothesized that the mitochondrial function in the skin and maintenance of tight junctions that play an important role in the skin barrier could be regulated by baicalein.

## 2. Materials and Methods

### 2.1. Materials

Baicalein and N-acetyl-1-cysteine (NAC) were purchased from Sigma Chemical Co. (St. Louis, MI, USA). Hydrogen peroxide 30% was purchased from Junsel Chemical Co. (Tokyo, Japan). 2′,7′-dichlorodihydrofluorescein diacetate (DCF-DA) was purchased from Invitrogen™ (Waltham, MA, USA). Antibodies against collagen type 1 alpha 1 (Col1A1) (ab34710), matrix metalloproteinase-1 (MMP-1) (ab38929), zonula occludens-1 (ZO-1) (ab96587), occludin (ab216327), and Nrf2(ab31163) were purchased from Abcam Inc (Cambridge, UK). PGC-1α (sc-518025), PINK1 (sc-517353), NQO-1 (sc-32793), claudin-4 (sc-376643), and β-actin (sc-47778) were purchased from Santa Cruz Biotechnology (Santa Cruz, CA, USA).

### 2.2. Cell Culture and H_2_O_2_ Treatment

Human epithelial keratinocytes HaCaT cells were obtained from American Type Culture Collection (ATCC, Manassas, VA, USA). Cells were cultured in Dulbecco’s modified Eagle’s medium (DMEM; Corning, cat. no. 10-013-CV) supplemented with 10% fetal bovine serum (FBS; Corning, cat. no. 35-015-CV) and 1% penicillin/streptomycin (P/S; Gibco, cat. no. 1780180) at 37 °C in a humidified 5% CO_2_ incubator. The cells were then pretreated with baicalein (20 or 40 μM) or N-acetyl-L-cysteine (NAC; 5 mM) in serum-free DMEM for 1 h. Then, H_2_O_2_ (500 μM) was added and the cells were cultured for 6 h. Baicalein was dissolved in dimethyl sulfoxide (DMSO) to prepare a 100 mM stock solution and stored at 4 °C. The final concentration of DMSO in the culture medium was <0.1% (*v*/*v*). H_2_O_2_ and NAC were diluted with DMEM.

### 2.3. Cell Viability Assay

Cell viability was assessed with MTS reagent (Promega™, G358C, Charbonnières les Bains, France), in accordance with the manufacturer’s instructions. Cells were seeded into 96-well cell culture plates and incubated for 24 h at 37 °C in 5% CO_2_. Next, treated with different concentrations of Baicalein (5 to 40 μM) and H_2_O_2_ (50 to 800 μM). Subsequently, the cells were incubated with 15 μL MTS reagent in DMEM at 37 °C for 2 h. Absorbance was measured at 490 nm, using a microplate reader (TriStar2-LB942, BERTHOLD, Bad Wildbad, Germany).

### 2.4. Measurement of Intracellular ROS Production

Levels of intracellular ROS were measured using the dichloro-dihydro-fluorescein diacetate (DCF-DA) fluorescent dye. HaCaT cells were seeded in 96-well culture plates and incubated for 24 h. Next, they were pretreated with baicalein (20 or 40 μM) or NAC (5 mM) for 1 h. Following the pretreatment, the cells were incubated with 10 μM of H2DCFDA (Invitrogen, cat. no. D399) for 30 min in the dark. Then, 500 μM H_2_O_2_ was added to the wells. The fluorescence intensity was measured at excitation and emission wavelengths of 485 and 535 nm, respectively, using a microplate reader.

### 2.5. Western Blot Analysis

Cells were lysed in NP40 lysis buffer (Invitrogen, Waltham, MA, USA) containing a protease inhibitor cocktail (GenDEPOT, Barker, TX, USA) on ice for 30 min. The protein concentration was quantified using an albumin standard (Thermo Scientific, Waltham, MA, USA). Equal amounts of proteins were resolved by sodium dodecyl sulfate polyacrylamide gel electrophoresis (SDS-PAGE) and transferred to polyvinylidene difluoride (PVDF) membranes (Merck Millipore, Darmstadt, Germany). The membranes were blocked at room temperature for 1 h with 3% bovine serum albumin (BSA) in TBS with tween 20. Following blocking, the membranes were incubated with primary antibodies (1:1000 dilution) for 16 h at 4 °C. The membranes were then washed thrice with TBST and incubated with the HRP-conjugated secondary antibodies followed by incubation with the enhanced chemiluminescence (ECL) solution (Dynebio, Seongnam, Korea). Proteins were visualized using an Amersham imager 600 (GE Healthcare, Chicago, IL, USA) and quantified with the ImageJ program.

### 2.6. RNA Extraction and RT-qPCR

Total RNA was extracted using the RNeasy Mini Kit (QIAGEN, Hilden, Germany) according to the manufacturer’s instructions. Total RNA was quantified using NanoDrop™ 2000 (Thermo Scientific, Waltham, MA, USA). The extracted RNA (1 µg) was reverse transcribed into cDNA. The expression of MMP-1, Col1A1, ZO-1, occludin, PGC-1α, PINK1, Nrf2, NQO-1, HO-1, and GAPDH was determined by RT-qPCR using TOPreal™ qPCR 2X Premix (enzynomics, Daejeon, Korea). All qPCR analyses were performed using a QuantStudio 1 Real-Time PCR Instrument (Thermo Scientific, Waltham, MA, USA). The relative expression level of the target genes was calculated using the ΔΔCt method using GAPDH as the internal control.

### 2.7. Soluble Collagen Assay

Total soluble collagen was measured using a Sircol™—Soluble Collagen Assay Kit (Biocolor Ltd., Carrickfergus, UK). A total of 200 µL of isolation and concentration reagent was added to 1 mL of conditioned medium from the cell culture in a tube and incubated at 4 °C for 15 min. Each tube was then vortexed thrice and further incubated at 4 °C for 15 min. The tubes were centrifuged at 13,000× *g* for 10 min and the supernatant was discarded. Sircol™ dye (1 mL) was added to the pellet and incubated for 30 min on a mechanical shaker. The tubes were centrifuged at 13,000× *g* for 10 min. The supernatant was discarded and 750 µL of acid-salt wash reagent was added to the pellet. The tubes were then centrifuged at 13,000× *g* for 10 min to pellet the collagen–dye complex. After removing the supernatant, 250 µL of Sircol™ alkali reagent was added to the pellet and vortexed. The relative absorbance was measured at 556 nm using a microplate reader.

### 2.8. Immunofluorescence

Cells were cultured in 4-well chamber slides. Following treatment, the cells were fixed with 4% formaldehyde for 20 min at room temperature. A 5% BSA + 0.5% Triton X-100 in PBS solution was added to the cells for 1 h to block non-specific sites. After blocking, the cells were incubated with rabbit anti-ZO-1 (1:600) and mouse anti-claudin-4 (1:300) primary antibodies at 4 °C overnight. Following washes with PBS, the cells were incubated with goat-anti-rabbit IgG Alexa Fluor 488 (Invitrogen) or goat-anti-mouse IgG Alexa Fluor 568 (Invitrogen) secondary antibodies (1:1000) for 1 h in the dark. Cells were stained with DAPI in mounting solution (Vector Laboratories, Newark, CA, USA) and analyzed with an Olympus BX53 fluorescence microscope (Olympus Corporation, Tokyo, Japan). Fluorescence intensities were calculated using the Image J program.

### 2.9. Enzyme-Linked Immunosorbent Assay (ELISA) for Detection of Parkin

A Parkin ELISA Kit (MyBioSource, MBS3802074, San Diego, CA, USA) was used according to the manufacturer’s instructions to detect Parkin levels. A total of 40 µL of diluent buffer and 100 µL of HRP-conjugate reagent was added to each well containing 10 µL of sample and incubated for 1 h at 37 °C. The wells were washed five times with 400 µL of wash solution followed by the addition of 50 µL each of the chromogen solutions A and B. The samples were protected from light and incubated at 37 °C for 15 min. Finally, 50 µL of stop solution was added to the wells, and the absorbance was measured at 450 nm using a microplate reader.

### 2.10. Measurement of the Cellular Oxygen Consumption Rate

Mitochondrial respiration was assessed using a Seahorse XFp analyzer (Agilent Technologies, Santa Clara, CA, USA). Cells were cultured in Seahorse XFp cell culture miniplate (Agilent Technologies). Following treatment, the medium was replaced with Seahorse XFp DMEM supplemented with 4.5 g/L d-glucose, l-glutamine, and sodium pyruvate and the plates were incubated for 30–60 min in a non-CO_2_ incubator at 37 °C. Finally, oligomycin (1.5 µM), FCCP (0.5 µM), and a mixture of rotenone and antimycin A (0.5 µM) were sequentially injected into the flux cartridge injection port according to the instrument setting procedure for calibration. Data analysis was performed following calibration.

### 2.11. Statistical Analysis

All data correspond to three independent experiments (*n* = 3). The values were compared using the Student’s *t*-test and are presented as mean ± standard deviation (S.D.).

## 3. Results

### 3.1. Effects of H_2_O_2_ and Baicalein on Intracellular ROS Production in HaCaT Keratinocytes

**Figure 1:**
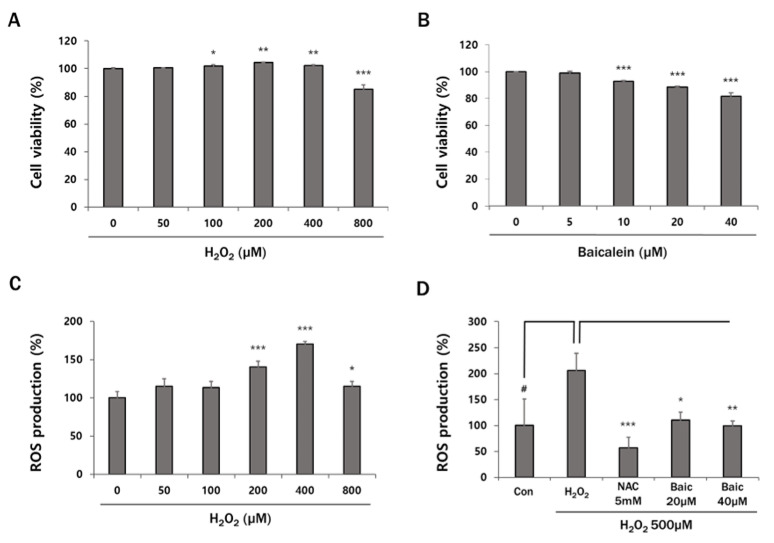
Baicalein reduces H_2_O_2_-induced ROS production. HaCaT cells were (**A**) treated with H_2_O_2_ (50~800 μM) and (**B**) baicalein (5~40 μM). After incubation for 24 h, MTS assay was performed, and cells were (**C**) treated with H_2_O_2_ (50~800 μM) and (**D**) pretreated with NAC (5 mM; positive control) or baicalein (20 and 40 μM*)* for 1 h prior to treatment with 500 μM H_2_O_2_ and intracellular ROS production was evaluated using the DCF-DA dye and a plate reader. The data represent the results of three independent experiments (*n* = 3) and are presented as the mean ± S.D. # *p* < 0.05 vs. control group; * *p* < 0.05, ** *p* < 0.01, *** *p* < 0.005 vs. H_2_O_2_-treated group.

To determine the concentration of H_2_O_2_ and baicalein, we performed the cell viability assay. They showed low cytotoxicity against HaCaT cells. As shown in Figure 1A, H_2_O_2_ between 50 and 400 μM did not induce cell death at all, but it was significantly reduced at 800 μM. Baicalein presented a reduction of 18% cell viability at 40 μM (Figure 1B). These results suggest that the subsequent alterations between them are not due to the cytotoxicity of baicalein. The antioxidant activity of baicalein on intracellular ROS produced in HaCaT cells was investigated using the DCF-DA fluorescent dye. The intracellular ROS production increased dose-dependently following treatment with various concentrations of H_2_O_2_. Treatment with 400 μM H_2_O_2_ resulted in a 70% increase in ROS levels compared with those in the control group. However, following treatment with 800 μM H_2_O_2_, the ROS levels decreased to a level similar to those in the control group (Figure 1C). We speculate that this may be due to H_2_O_2_-induced toxicity in HaCaT keratinocytes. Therefore, we treated the cells with 500 μM of H_2_O_2_ and baicalein in the subsequent experiments. As shown in Figure 1D, ROS production increased significantly by 106% in the H_2_O_2_-treated group compared with that in the control group. Treatment with baicalein effectively inhibited the intracellular ROS production when compared with the H_2_O_2_-treated group. Similarly, NAC, a well-known antioxidant, reduced H_2_O_2_-induced ROS production. These results suggest that baicalein decreases intracellular ROS production in H_2_O_2_-treated HaCaT keratinocytes.

### 3.2. Effects of Baicalein on Collagen Degradation

**Figure 2:**
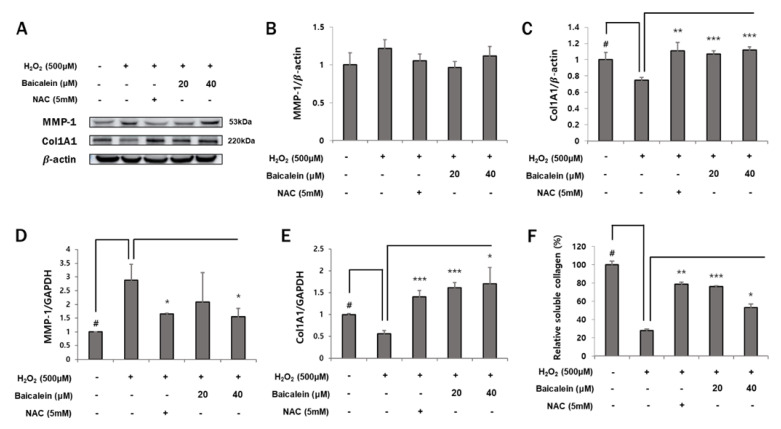
Baicalein inhibits H_2_O_2_-induced collagen degradation. HaCaT cells were pretreated with NAC (5 mM) or baicalein (20 and 40 μM) for 1 h, and then co-treated with 500 μM H_2_O_2._ (**A**–**C**) MMP-1 and Col1A1 protein levels were determined by Western blotting and analyzed using Image J. The protein levels were normalized to those of β-actin. (**D**,**E**) MMP-1 and Col1A1 mRNA levels were determined by RT-qPCR analysis. The mRNA levels were normalized to those of GAPDH. (**F**) Total soluble collagen levels were determined after 24 h of culture. The data represent the results of three independent experiments (*n* = 3), and are presented as the mean ± S.D. # *p* < 0.05 vs. control group; * *p* < 0.05, ** *p* < 0.01, *** *p* < 0.005 vs. H_2_O_2_-treated group.

Collagen is a major component of the ECM and is degraded by matrix metalloproteinases (MMPs). An increase in ROS production induces the expression of MMPs, which promotes the formation of wrinkles in the skin [34,35]. To evaluate the effect of baicalein on H_2_O_2_-mediated collagen degradation, MMP-1 and collagen levels were determined. As shown in Figure 2A,B,D, MMP-1 expression was increased following treatment with 500 μM H_2_O_2_. However, the MMP-1 protein level was attenuated following pretreatment with 20 and 40 μM baicalein (1.21 ± 0.1% vs. 0.96 ± 0.08%, 1.12 ± 0.1%, respectively). In addition, treatment with 40 μM baicalein caused a significant decrease in MMP-1 mRNA expression (2.89 ± 0.57% vs. 1.54 ± 0.31%). Similarly, baicalein showed an inhibitory effect on collagen degradation (Figure 2C,E). These results suggest that H_2_O_2_ induces degradation of Col1A1, which is significantly attenuated by treatment with 20 and 40 μM baicalein at both the protein and mRNA levels (0.75 ± 0.03% vs. 1.07 ± 0.04%, 1.12 ± 0.04%; 0.56 ± 0.07% vs. 1.62 ± 0.12%, 1.71 ± 0.37%, respectively). Furthermore, the total soluble collagen level in the baicalein-treated group increased by approximately 2–3 fold when compared with that in the only H_2_O_2_-treated group (Figure 2F). Thus, baicalein may enhance the cell–matrix junction by attenuating the expression of MMP-1 and preventing Col1A1 degradation.

### 3.3. Baicalein Prevents Cellular Tight Junction Disruption

**Figure 3:**
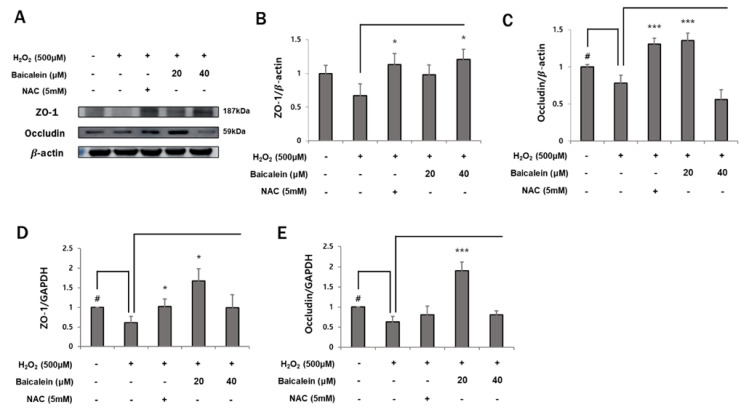

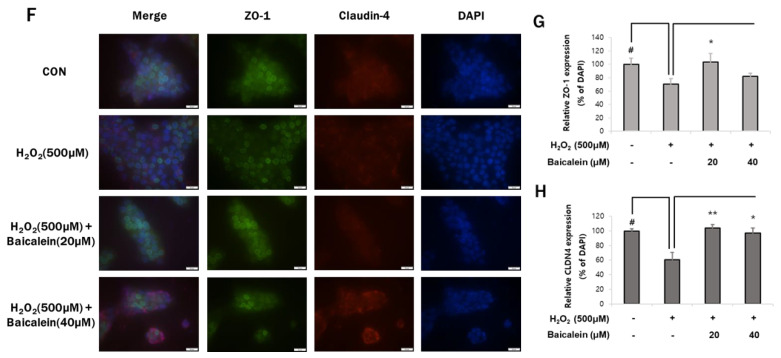
Effect of baicalein on cellular tight junctions in H_2_O_2_-treated HaCaT keratinocytes. (**A**–**C**) The expression of ZO-1 and occludin was determined by Western blotting and analyzed by Image J. The protein levels were normalized to those of β-actin. (**D**,**E**) ZO-1 and occludin mRNA levels were analyzed by RT-qPCR and normalized to those of GAPDH. (**F**) Expression of ZO-1 and occludin proteins in H_2_O_2_- and baicalein-treated HaCaT keratinocytes were visualized by immunofluorescence: ZO-1 (green), claudin-4 (red), and DAPI (blue). (**G**,**H**) Mean fluorescence intensities of ZO-1 and claudin 4 were measured using Image J. Each graph represents the relative value compared to that in control. The data represent the results of three independent experiments (*n* = 3) and are presented as the mean ± S.D. # *p* < 0.05 vs. control group; * *p* < 0.05, ** *p* < 0.01, *** *p* < 0.005 vs. H_2_O_2_-treated group.

An increased intracellular ROS level induces disruption of the cellular tight junctions [10]. Our previous results demonstrated that pretreatment with baicalein inhibits H_2_O_2_-induced ROS production. Therefore, we hypothesized that baicalein might also have a protective effect on the H_2_O_2_-induced disruption of tight junctions. We investigated the effects of baicalein on cellular tight junctions in H_2_O_2_-treated HaCaT keratinocytes. As shown in Figure 3A–C, treatment with 500 μM H_2_O_2_ resulted in an approximately 30% decrease in ZO-1 and occludin protein levels compared with those in the control group (ZO-1 1 ± 0.12% vs. 0.67 ± 0.17%; occludin 1 ± 0.03% vs. 0.78 ± 0.11%, respectively). However, pretreatment of 20 μM baicalein restored the protein expression of ZO-1 and occludin (ZO-1 0.67 ± 0.17% vs. 0.98 ± 0.15%; occludin 0.78 ± 0.11% vs. 1.35 ± 0.1%, respectively). In fact, the mRNA expression of these tight junction proteins was visibly increased following the treatment of 20 μM baicalein when compared to the H_2_O_2_-treated group (ZO-1 0.61 ± 0.16% vs. 1.68 ± 0.31%; occludin 0.63 ± 0.14% vs. 1.90 ± 0.21%, respectively) (Figure 3D,E). Additionally, as shown in Figure 3F, immunofluorescence analysis confirmed the baicalein-mediated recovery of the expression of ZO-1 and occludin. All images were acquired at the same fluorescence intensity. The expression of ZO-1 and claudin-4 was significantly decreased by H_2_O_2_, but the treatment of baicalein restored it to a level almost similar to that in the control group. These results demonstrate the potential of baicalein as a strong defense mechanism against ROS-induced disruption of tight junctions.

### 3.4. Baicalein Helps to Maintain Mitochondrial Homeostasis

**Figure 4:**
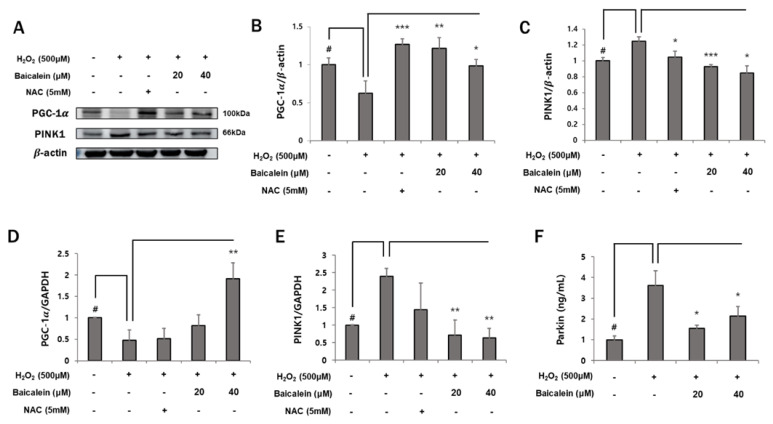
Baicalein maintains mitochondrial homeostasis during H_2_O_2_-induced mitochondria dysfunction. HaCaT cells were pretreated with NAC (5 mM) or baicalein (20 and 40 μM) for 1 h, and then co-treated with 500 μM H_2_O_2_. The expression of PGC-1α and PINK1 was evaluated by Western blotting and RT-qPCR. (**A**) PGC-1α and PINK1 proteins were detected by Western blotting, and (**B**,**C**) protein levels of PGC-1α and PINK1 were quantitated using Image J and normalized to those of β-actin. (**D,E**) PGC-1α and PINK1 mRNA levels were analyzed by RT-qPCR and normalized to those of GAPDH. (**F**) The effect of baicalein on Parkin expression was analyzed by ELISA. Parkin protein concentration in HaCaT keratinocyte culture medium was measured at 450 nm using a plate reader. The data represent the results of three independent experiments (*n* = 3) and are presented as the mean ± S.D. # *p* < 0.05 vs. control group; * *p* < 0.05, ** *p* < 0.01, *** *p* < 0.005 vs. H_2_O_2_-treated group.

ROS are known to induce mitochondrial dysfunction [9]. The enhancement of tight junctions may improve mitochondrial function. We evaluated mitochondrial biogenesis and mitophagy to determine whether baicalein improves mitochondrial function. As shown in Figure 4A,B,D, PGC-1α, a regulator of mitochondrial biogenesis, was reduced following H_2_O_2_ treatment at both protein and mRNA levels (1 ± 0.09% vs. 0.63 ± 0.16%; 1 ± 0.01% vs. 0.47 ± 0.24%, respectively). However, its level was significantly restored following treatment with 40 μM baicalein (0.62 ± 0.16% vs. 0.99 ± 0.08%; 0.47 ± 0.24% vs. 1.92 ± 0.37%, respectively). In addition, mitochondrial biogenesis increased significantly in the baicalein group compared with that in the control group. In addition, H_2_O_2_ treatment significantly increased PINK1 level, which was inhibited by pretreatment with 20 μM and 40 μM baicalein at both the protein and mRNA levels (1.25 ± 0.07% vs. 0.93 ± 0.03%, 0.85 ± 0.11%; 2.4 ± 0.23% vs. 0.72 ± 0.43%, 0.63 ± 0.27%, respectively) (Figure 4C,E). ELISA for the expression of Parkin, another mitophagy marker, showed similar results as PINK1 (Figure 4F). The data showed that the H_2_O_2_-mediated decrease in mitochondrial biogenesis and increase in mitophagy were reversed by baicalein treatment. These data suggest that baicalein protects HaCaT keratinocytes from H_2_O_2_-induced mitochondrial dysfunction.

### 3.5. Baicalein Inhibits OCR Reduction

**Figure 5:**
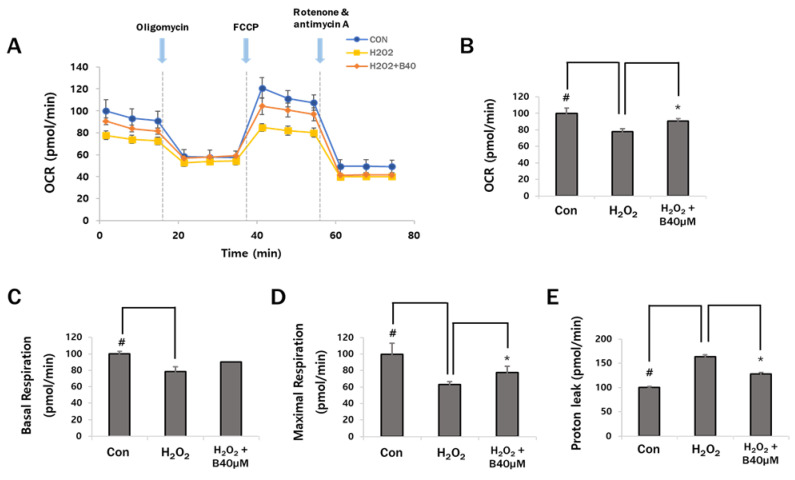
The effects of H_2_O_2_ and baicalein on mitochondrial respiration were measured by Seahorse XFp analyzer. The cells were pretreated with 40 μM baicalein for 1 h and co-treated with 500 μM H_2_O_2_ for 6 h. (**A**,**B**) Oxygen consumption rates of H_2_O_2_ and baicalein in HaCaT keratinocytes. (**C**) Basal respiration, (**D**) maximal respiration, (**E**) proton leak was measured simultaneously with OCR measurements. Each data represent 3 independent experiments (*n* = 3), and the values are represented as the mean ± S.D. # *p* < 0.05 vs. control group and * *p* < 0.05 vs. H_2_O_2_-only-treated group.

One of the most accurate ways to determine mitochondrial function is to measure cellular respiration, as it directly reflects any impairment in the electron transport chain. OCR measurements reveal mitochondrial function and ATP synthesis capacity [36]. Our previous results demonstrated that pretreatment with baicalein provided cytoprotection via the inhibition of ROS production and mitochondrial dysfunction. Therefore, we hypothesized that baicalein might restore the H_2_O_2_-mediated reduction in mitochondrial respiration. OCR was analyzed using the Seahorse XFp analyzer. Oligomycin was used to inhibit ATP synthase (complex V) in the electron transport chain and reduce mitochondrial respiration. FCCP, a protonophore, disrupts the inner membrane gradient by transporting protons across the inner mitochondrial membrane. Rotenone (complex I inhibitor) and antimycin A (complex III inhibitor) block the function of the electron transport chain. As shown in Figure 5A, H_2_O_2_ treatment decreased OCR, which was restored by 20% following treatment with 40 µM baicalein. Detailed analysis revealed that the OCR, basal respiration, and maximal respiration were increased following treatment with 40 µM baicalein compared to treatment with H_2_O_2_ alone (77.7 ± 3.8% vs. 90.5 ± 3.0%; 78.7 ± 3.2% vs. 90.4 ± 5.6%; 62.9 ± 3.6% vs. 77.8 ± 7.3%, respectively). The increase in OCR and maximal respiration were statistically significant (Figure 5B,D). and basal respiration also increased (Figure 5C). Proton leak was significantly decreased by 36% following treatment with 40 µM baicalein compared to treatment with H_2_O_2_ alone (163.4 ± 4.1% vs. 127.8 ± 2.7%) (Figure 5E). Decreased basal and maximal respiration and increased proton leak are indicators of mitochondrial dysfunction [37]. These data suggest that H_2_O_2_ treatment induced OCR reduction, whereas baicalein treatment provided cytoprotection by improving mitochondrial function.

### 3.6. Baicalein Regulates Antioxidant Enzymes in HaCaT Keratinocytes

**Figure 6:**
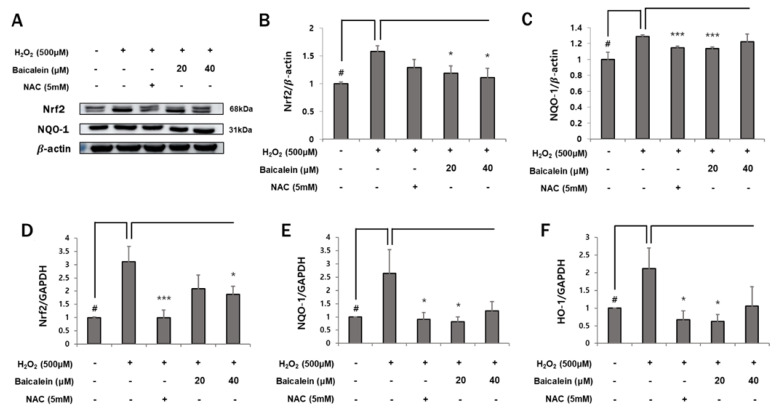
Baicalein mediates cytoprotective effects by regulating the Nrf2/NQO-1/HO-1 pathway. (**A**–**C**) The expression of antioxidant enzymes was determined by Western blotting and analyzed by Image J. The protein levels were normalized to those of β-actin. (**D**–**F**) Nrf2, NQO-1, and HO-1 mRNA levels were analyzed by RT-qPCR and normalized to those of GAPDH. The data represent the results of three independent experiments (*n* = 3) and are presented as the mean ± S.D. # *p* < 0.05 vs. control group; * *p* < 0.05, *** *p* < 0.005 vs. H_2_O_2_-treated group.

Nrf2 is an important regulator of ROS-induced mitochondrial damage and represents a major pathway regulated by oxidative stress. Nrf2 regulates the expression of antioxidant enzymes such as HO-1 and NQO-1. In addition, Nrf2 maintains mitochondrial homeostasis [38]. Therefore, we sought to determine whether the cytoprotective effects of baicalein were related to the activation of the Nrf2 pathway. We found that baicalein regulates the Nrf2 pathway. The protein expression of Nrf2 and NQO-1 was significantly higher in the H_2_O_2_-treated group compared with that in the control group (Nrf2 1 ± 0.03% vs. 1.58 ± 0.1%; NQO-1 1 ± 0.09% vs. 1.29 ± 0.02%, respectively). However, baicalein restored the levels of the antioxidant proteins to those in the control group. Specifically, treatment with 20 μM baicalein significantly decreased the levels of both Nrf2 and NQO-1 (Nrf2 1.58 ± 0.1% vs. 1.18 ± 0.13%; NQO-1 1.29 ± 0.02% vs. 1.14 ± 0.02%, respectively). The expression of HO-1 was undetectable by Western blotting (Figure 6A–C). The RT-qPCR results for Nrf2 and NQO-1 were similar to those of Western blotting. Additionally, the mRNA expression of HO-1 was significantly decreased following treatment with 20 μM baicalein compared with the H_2_O_2_-treated group (2.1 ± 0.58% vs. 0.63 ± 0.19%) (Figure 6D,E). These data suggest that the cytoprotective effects of baicalein in HaCaT keratinocytes are mediated through the maintenance of mitochondrial homeostasis via regulation of the Nrf2/NQO-1/HO-1 pathway.

## 4. Discussion

In the present study, we evaluated the antioxidant activity of baicalein in HaCaT cells under conditions of H_2_O_2_-induced oxidative stress and studied its effects on tight junction proteins and mitochondrial homeostasis. This is the first study to reveal the regulation of tight junctions related to the maintenance of mitochondrial homeostasis and the skin barrier.

ROS regulate cell signaling and maintains the homeostasis [39]. ROS are maintained at low and fixed basal levels in normal cells. However, the exposure of cells to external stimuli, such as UVR, results in the accumulation of excessive ROS, which causes oxidative stress and damages the skin [40,41]. Several studies have reported that continuous ROS accumulation leads to mtDNA mutations, mitochondria dysfunction, and the disruption of cellular homeostasis. Aberrant removal of ROS leads to decreased skin function and aging [22,23,42]. Therefore, the recovery of cellular homeostasis through the inhibition of ROS production effectively reduces oxidative-stress-induced skin aging.

In our experiments, treatment with H_2_O_2_ induced oxidative stress in the cells. H_2_O_2_ increased intracellular ROS production and MMP-1 expression. Accordingly, the expression of Col1A1 was decreased. In addition, the expression of tight junction proteins, such as ZO-1, occludin, and claudin-4, was decreased in the H_2_O_2_-treated group. The expression of PGC-1α was inhibited, whereas that of mitophagy-related proteins, such as PINK1 and Parkin was increased. In addition, mitochondrial respiration was also decreased following H_2_O_2_ treatment. The expression of Nrf2, antioxidant transcription factor, and antioxidant enzymes, including NQO-1, and HO-1, was increased in response to oxidative stress. In contrast, the expression of these proteins was reversed following treatment with baicalein.

First, we evaluated the antioxidant activity of baicalein. The IC_50_ value of baicalein against H_2_O_2_-induced ROS production was 46 µM. Meanwhile, 20 µM and 40 µM of baicalein corresponded to IC_45_ and IC_49.4_, respectively. There was no significant difference according to the concentration of baicalein. Thus, we focused not only on IC_50_ for ROS production, but also on investigating whether baicalein has a varying cytoprotective effect in a dose-dependent manner. Baicalein significantly decreased H_2_O_2_-induced intracellular ROS production in HaCaT keratinocytes (Figure 1D). As a result, baicalein prevents skin aging by scavenging ROS [43].

The ECM is composed of fibrous proteins, such as collagens and elastin. The ECM regulates many cellular processes, including tissue growth, differentiation, and homeostasis [44]. Type I collagen is the most abundant structural protein in the skin and is primarily degraded by MMP-1. MMP-1 is rarely expressed under normal physiological conditions; however, its levels increase markedly in response to stress [34,45]. Tight junctions are important intercellular junctions that create a skin barrier and maintain cell polarity. They are also involved in maintaining the epithelial homeostasis [14,15]. Thus, both ECM and tight junction proteins are important for preventing skin aging. Therefore, we assessed the expression of ECM and tight junction proteins to evaluate the effect of baicalein on the maintenance of epithelial homeostasis. A previous study demonstrated that treatment with H_2_O_2_ increases the MMP-1 expression [46]. In the current study, we found that baicalein effectively reduced both the protein and mRNA expression of MMP-1. Concomitantly, the expression of Col1A1 was increased (Figure 2). A previous study reported that baicalein might improve the skin barrier function [47]. In our study, baicalein effectively increased the protein and mRNA expression of the tight junction proteins. Additionally, we confirmed the increase in tight junction proteins using immunofluorescence. Treatment with 20 μM baicalein significantly increased the expression of tight junction proteins (Figure 3). These results suggest that baicalein strengthens epithelial homeostasis and protects against oxidative stress.

Mitochondria play an important role in the skin. Mitochondrial homeostasis is maintained via the regulation of mitochondrial biogenesis and mitophagy. A balance between these two processes is essential for maintaining healthy mitochondria. Inadequate removal of ROS-induced mutations in the mtDNA may ultimately result in cell death. Thus, mitochondrial dysfunction may reduce skin function and cause aging [9,21,22,23]. ROS activate mitophagy and inhibit mitochondria biogenesis, resulting in an imbalance [48,49]. A previous study reported that baicalein restores the loss of mitochondrial membrane potential caused by oxidative stress in the HaCaT keratinocytes [50]. However, there are no reports on the cytoprotective effect of baicalein on mitochondrial biogenesis and mitophagy. As shown in Figure 4, baicalein increased the expression of PGC-1α and reduced that of PINK1 and Parkin. These results suggest that baicalein helps to restore HaCaT keratinocytes to a normal state and protects them against oxidative-stress-induced mitochondrial imbalance.

Studies have shown that the oxidative phosphorylation (OXPHOS) pathway generates most of the ATP required for active metabolism and the proliferation of epidermal cells [8]. We measured the mitochondrial respiratory function in the cells using the Seahorse XFp analyzer. Damaged mitochondria or electron receptors are known to lower the OCR. As shown in Figure 5, baicalein restored the decrease in OCR caused by H_2_O_2_. Baicalein also increased basal and maximal respiration, whereas it significantly decreased proton leak. The difference between the basal and maximal respiratory rate is called the reserve respiratory capacity (SRC). SRC is the amount of extra ATP that OXPHOS can produce in case of a sudden increase in energy demand, which is considered an extra capacity for cells to produce energy in response to increased stress [51]. In SRC, the 10% increase by baicalein compared to H_2_O_2_ may indicate a restoration of mitochondrial energy metabolism. Thus, ROS-induced mitochondrial dysfunction was restored by baicalein. Therefore, baicalein has a protective effect against H_2_O_2_-induced mitochondrial damage.

The Nrf2/ARE signaling cascade plays a major role in protecting cells by maintaining intracellular homeostasis and inducing the expression of oxidative-stress-responsive genes [28]. Nrf2 is activated in response to excessive oxidative stress [52]. Activated Nrf2 interacts with antioxidant response elements (AREs) to promote the transcription of endogenous antioxidant enzymes [29,30]. The restoration of Nrf2 to an equilibrium state lowers ROS and oxidative stress. As shown in Figure 6, the antioxidant enzymes such as Nrf2, NQO-1, and HO-1 were restored to control levels following baicalein treatment. This suggests that baicalein stabilizes the antioxidant enzymes overexpressed in response to oxidative stress. This is similar to the result of a previous study that demonstrated that NAC reverses H_2_O_2_-induced Nrf2 overexpression in MC3T3-E1 cells [53].

## 5. Conclusions

Baicalein exhibits antioxidant effects in response to H_2_O_2_-induced oxidative stress in HaCaT keratinocytes. Baicalein helps to maintain overall homeostasis by protecting against epithelial disruption, maintaining mitochondrial homeostasis, and increasing mitochondrial respiration.

## Data Availability

Data is contained within the article.
